# Addressing glenoid defects with distal clavicle autograft in revision total shoulder arthroplasty

**DOI:** 10.1016/j.xrrt.2024.10.006

**Published:** 2024-11-27

**Authors:** Ryan P. Bialaszewski, Ross Chapel, Frank Gerold

**Affiliations:** a University of Texas Rio Grande Valley, School of Medicine, Edinburg, TX, USA; b Rio Grande Bone and Joint, Rio Grande Regional Hospital, McAllen, TX, USA; c University of Texas Rio Grande Valley, Department of Orthopedics and Sports Medicine, School of Medicine, Edinburg, TX, USA

**Keywords:** Glenoid bone loss, Glenoid defect, Revision shoulder arthroplasty, Bone graft, Reverse total shoulder arthroplasty, Distal clavicle

Reverse total shoulder arthroplasty (rTSA) is indicated for commonly encountered conditions such as degenerative arthritis in the setting of rotator cuff arthropathy, inflammatory arthropathy, proximal humerus fractures, and prior failed shoulder arthroplasty. Indications for rTSA have continually expanded since its adoption in North America in 1990 and are associated with good outcomes.^2^, ^3^, ^4^^,^^16^ rTSA's benefits are primarily due to biomechanical advantages by moving the center of rotation of the humeral head more medial and inferiorly, allowing the deltoid to act on a longer fulcrum in the absence of a functional rotator cuff.

Despite promising clinical outcomes, rTSA involves complexities that can be particularly demanding for surgeons. The complexity increases exponentially in revision shoulder arthroplasty, primarily in the setting of infection, glenoid bone defects, and other deformities. Although a broad range of etiologies requires revision rTSA, this article focuses on central glenoid bone defects, which can be a common occurrence in revision surgery following previous total shoulder arthroplasty (TSA).^7^^,^^15^ These defects can be due primarily to previously failed shoulder arthroplasty, repetitive instability, inflammatory arthropathy, or hardware-induced glenoid wear. Despite the underlying etiology of revision rTSA, the primary goal is to replace or revise the glenoid and, or humeral components to improve both function and pain.

Appropriate evaluation and management of glenoid defects are essential for good clinical outcomes. Antuna previously described the classification of glenoid bone defects, categorizing between central, peripheral (anterior or posterior), or combined, with mild, moderate, and severe defects.^1^ Figure 1 below provides a visual representation of the Antuna classification.^5^ Severity is determined by the degree of involvement of the glenoid defect, with mild, involving less than one-third of the glenoid surface or rim, moderate, one to two-thirds, or severe if the defect was greater than two-thirds of the glenoid surface or rim.^1^ Reimplanting the glenoid component with or without bone grafting for mild and moderate defects is acceptable. However, severe central or combined deficiencies often preclude the implantation of a new glenoid component.^1^

**Figure 1:**
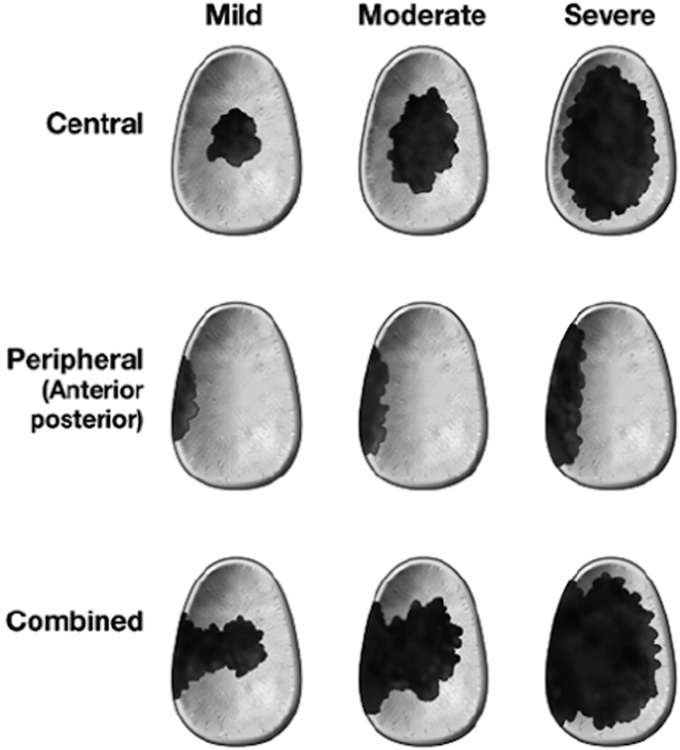
Glenoid Defects. Reproduced with permission from Flurin et al.^5^

Prior studies have estimated the prevalence of large glenoid defects requiring grafting in the setting of revision arthroplasty to be less than 10%, and the requirement for complex grafts is even rarer.^9^ According to several author recommendations, the threshold for glenoid defect size requiring grafting is typically when defects exceed 20% to 25% of the surface area.^12^^,^^15^^,^^18^ Glenohumeral stability can be compromised with defects larger than 25%.^8^^,^^10^ Because of this, glenoid component revision can be challenging to achieve with large defects. A recent biomechanical study by Formaini et al noted that adequate glenoid baseplate fixation requires at least 50% coverage to limit hardware micromotion, which can lead to loosening and failure.^6^ In addition, appropriate fixation of a glenoid component has a higher degree of satisfaction than those undergoing glenoid component removals.^1^

Management of large glenoid defects can occur in either a single-stage or two-stage technique. Two-stage techniques are typically reserved for advanced concentric and eccentric glenoid bone defects when a long-peg glenoid baseplate does not purchase at least 50% of its length in the native scapula.^9^ The goal of a 2-stage surgical technique seen in large glenoid defects is to minimize the chances of malunion or nonunion by not loading the glenoid, allowing it to heal with autologous cortical graft before baseplate insertion.

The inherent drawbacks of staged procedures include the necessity for two distinct surgeries, thereby amplifying the likelihood of prosthetic joint infection and other associated complications. Adopting a 1-stage surgical approach mitigates the risks associated with morbidity from a second surgery. In revision surgery, common autograft harvest sites include the iliac crest and proximal tibia, given the unavailability of the humeral head. However, this alternative introduces the potential risk of incomplete resorption of the glenoid graft component, posing a risk of loosening within the glenoid component. Furthermore, the necessity of having a second surgical site comes with its own inherent risks related to donor site morbidity and infection.

This article describes a case report performed as a single-stage technique to reconstruct a large, central glenoid bone defect in revision shoulder arthroplasty utilizing the distal clavicle. An autologous distal clavicle bone graft is combined with a reverse total shoulder glenoid implant to reconstruct the glenoid bone defect. The deltopectoral incision is carried superiorly to harvest the distal clavicle bone grafting for subsequent graft impaction and glenoid baseplate insertion, allowing for stability and realignment of the glenohumeral joint.

## Materials and methods

### History/preoperative planning

**Figure 2:**
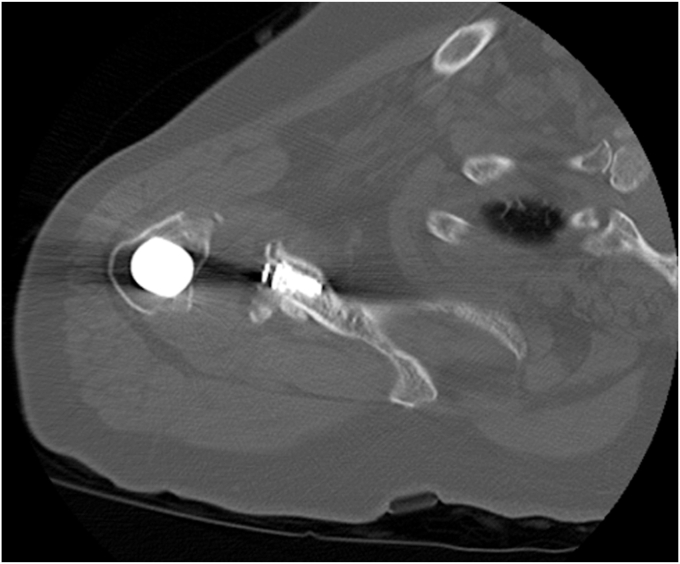
Preoperative CT showing aseptic loosening of prior total shoulder arthroplasty. *CT*, computed tomography.

**Figure 3:**
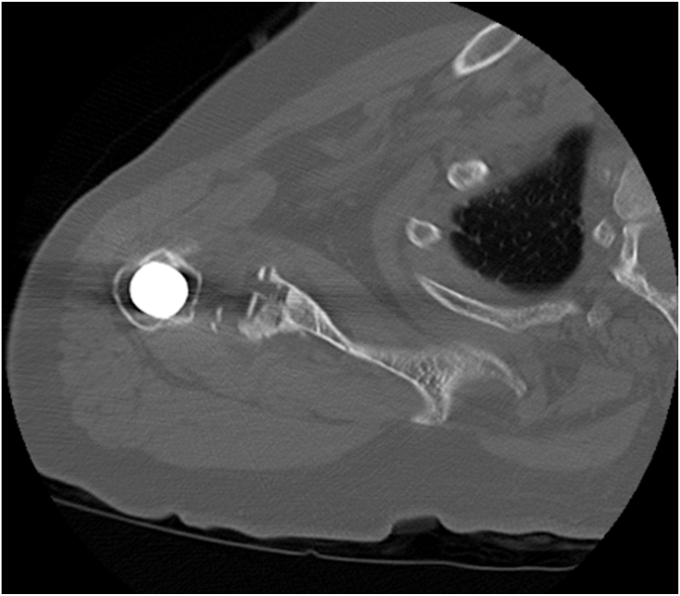
Preoperative axial *CT* demonstrating a glenoid defect suggesting aseptic loosening of prior total shoulder arthroplasty. *CT*, computed tomography.

**Figure 4:**
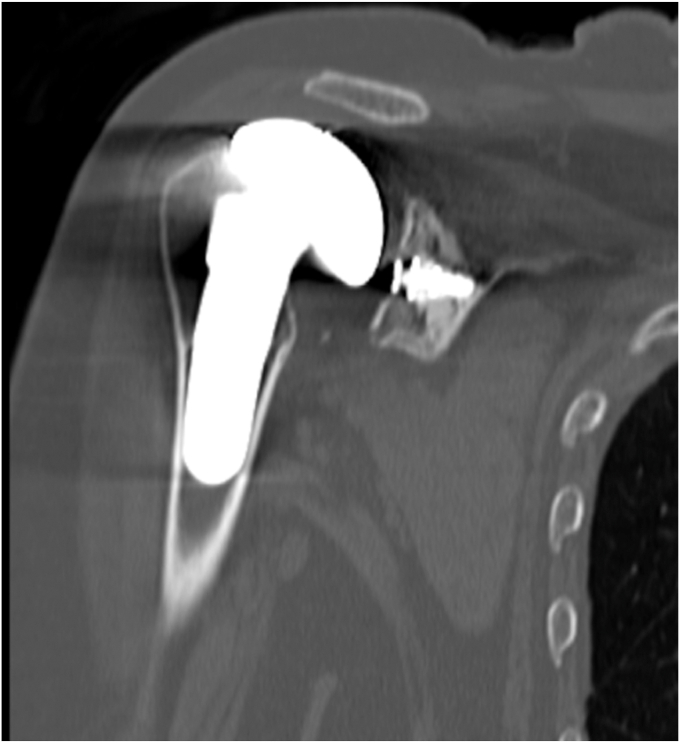
Preoperative CT showing aseptic loosening of prior total shoulder arthroplasty. *CT*, computed tomography.

The patient was a female aged 67 years who presented with concerns of right shoulder pain in the setting of prior TSA performed at an outside institution. Despite attempts at conservative therapy, including physical therapy, nonsteroidal anti-inflammatory medications, and corticosteroid injection, her symptoms persisted, and was referred for evaluation. It was noted her activities of daily living (ADLs) were severely limited due to the refractory nature of her symptoms. On examination, her active and passive forward elevation was 45 degrees with severe pain. External rotation was also diminished, measured at 50 degrees. Given a negative external rotation lag sign, infraspinatus and supraspinatus tendon integrity was suspected to be intact. Computed tomography was obtained and consistent with aseptic hardware loosening with some calcar stress shielding, as seen in Figure 2, Figure 3, Figure 4. Laboratory analysis, which included a complete blood count, sedimentation rate, and C-reactive protein, was within normal limits and not concerning for an underlying infectious process. Given the patient's presentation, physical exam findings, and imaging reports, a reverse TSA revision was planned.

### Surgical technique

The anesthesia team performed a regional interscalene block before induction. The patient was then induced and intubated accordingly. A beach chair position was then obtained, and all bony prominences were padded and protected. The patient's right upper extremity was prepped and draped in a standard sterile fashion, and appropriate time-out measures were taken.

Following a time-out, a standard deltopectoral approach was used, following the scar plane down the deltopectoral interval used in the patient's prior TSA. Extensive scarring of the deltoid and subdeltoid spaces required lysis of adhesions before deltoid mobilization with neuromonitoring, protecting the axillary nerve. Subscapularis was then tagged and mobilized using a peel approach. Upon opening the rotator interval, we obtained synovial fluid cultures and an intraoperative frozen section, which showed no signs of active infection.

**Figure 5:**
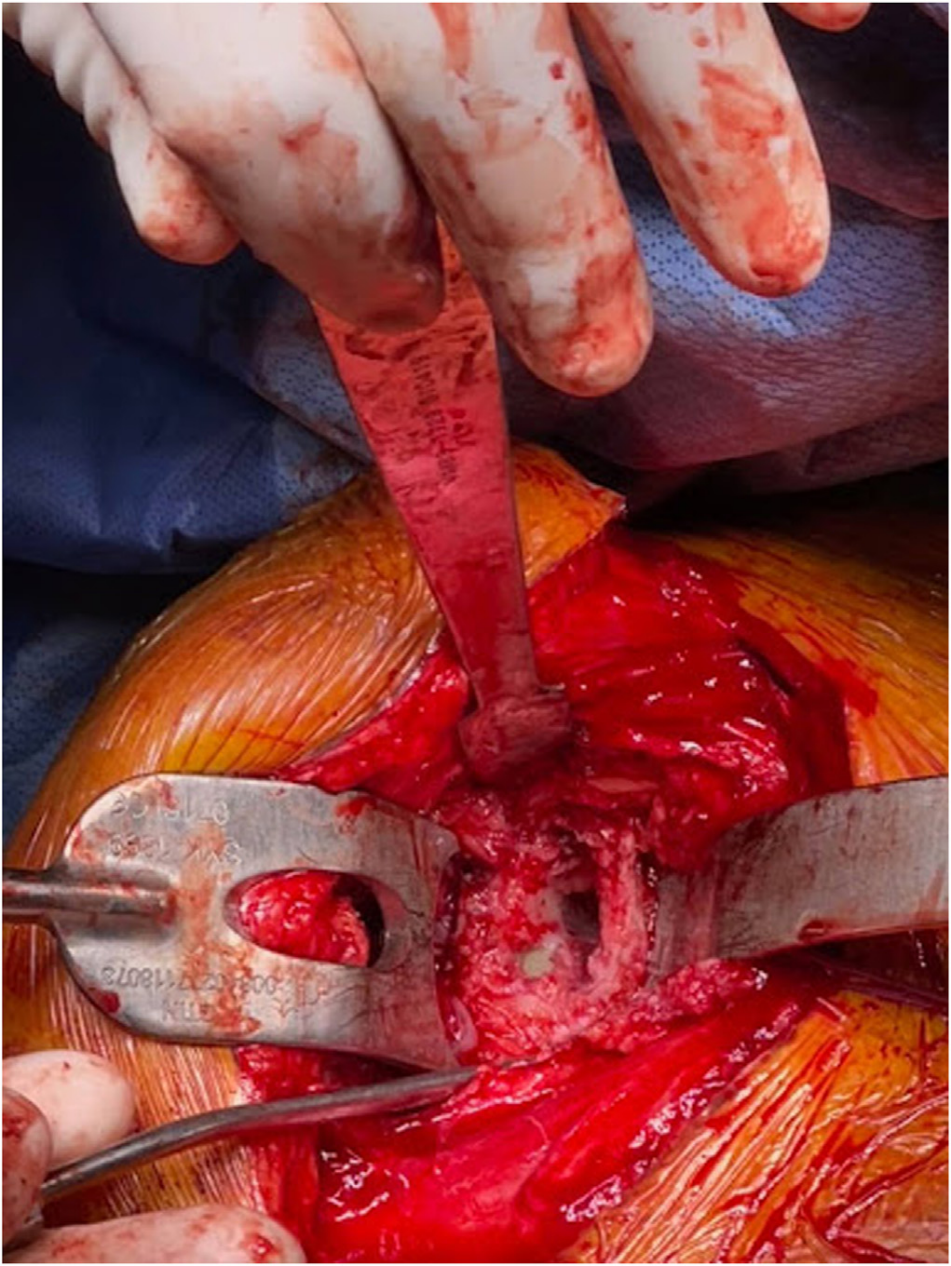
Large central glenoid defect following the removal of the glenoid baseplate stem.

**Figure 6:**
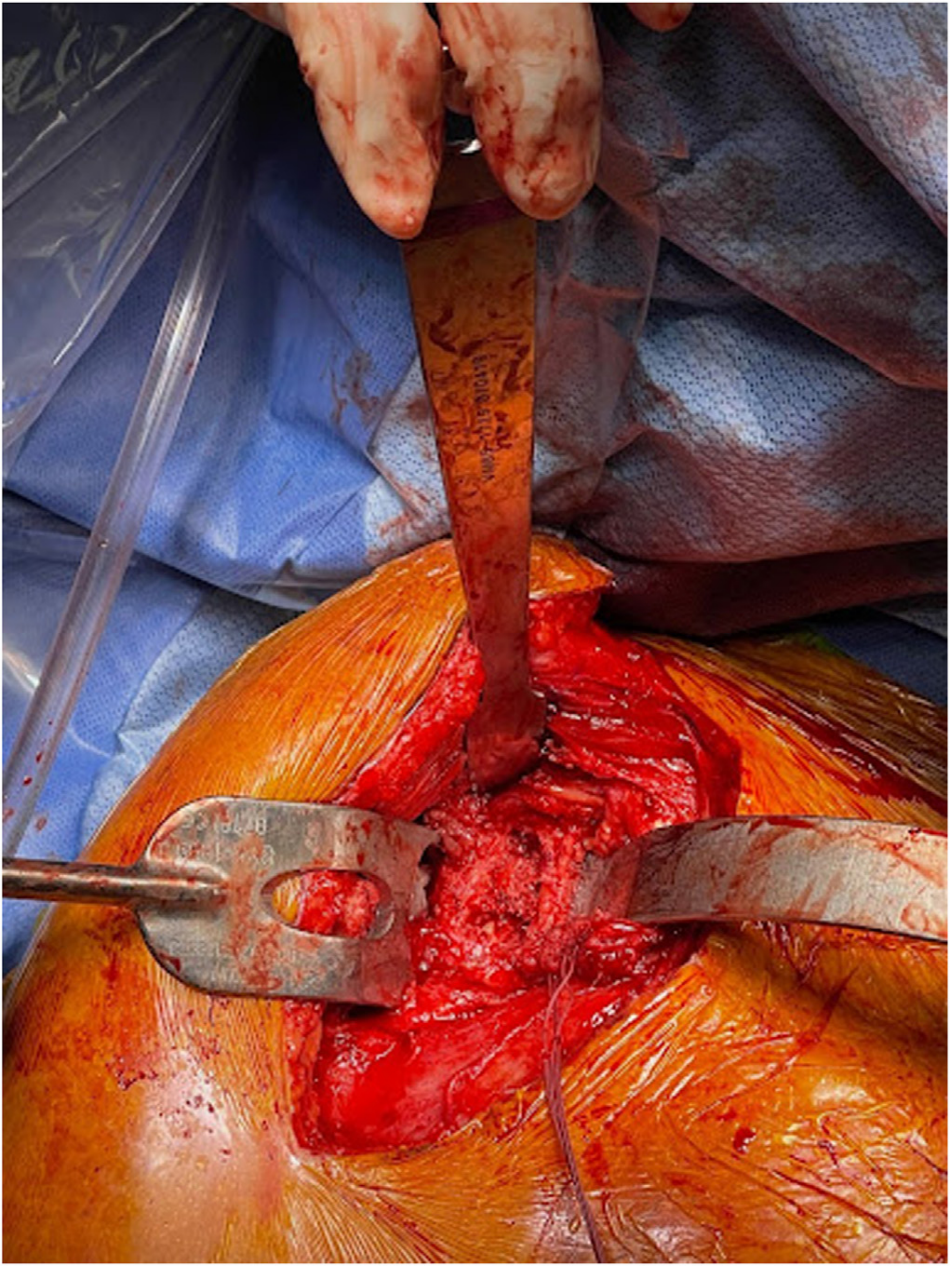
Large central glenoid defect following autologous clavicle graft impaction.

**Figure 7:**
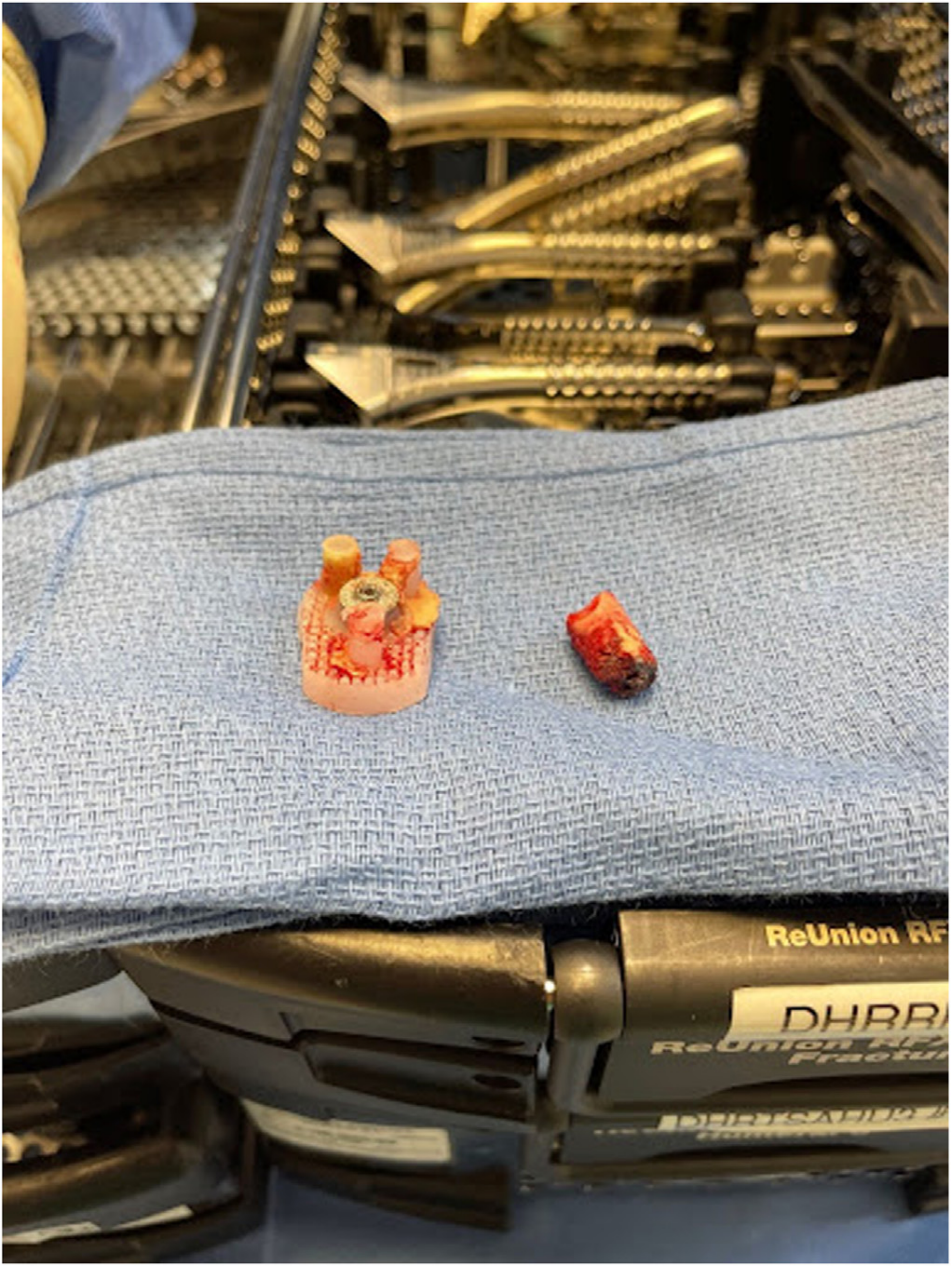
Trabeculated glenoid baseplate with fractured stem.

**Figure 8:**
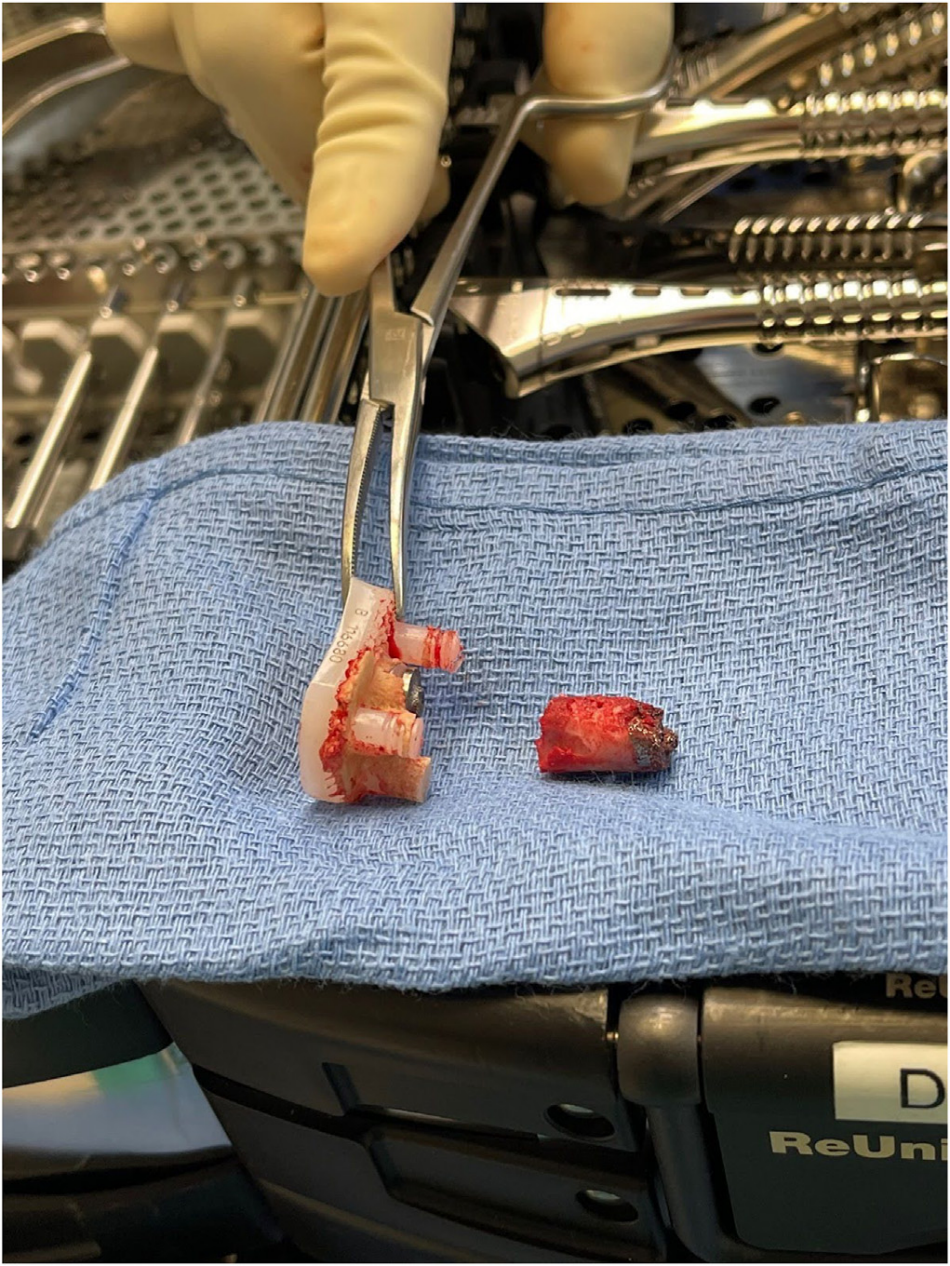
Trabeculated glenoid baseplate with fractured stem.

**Figure 9:**
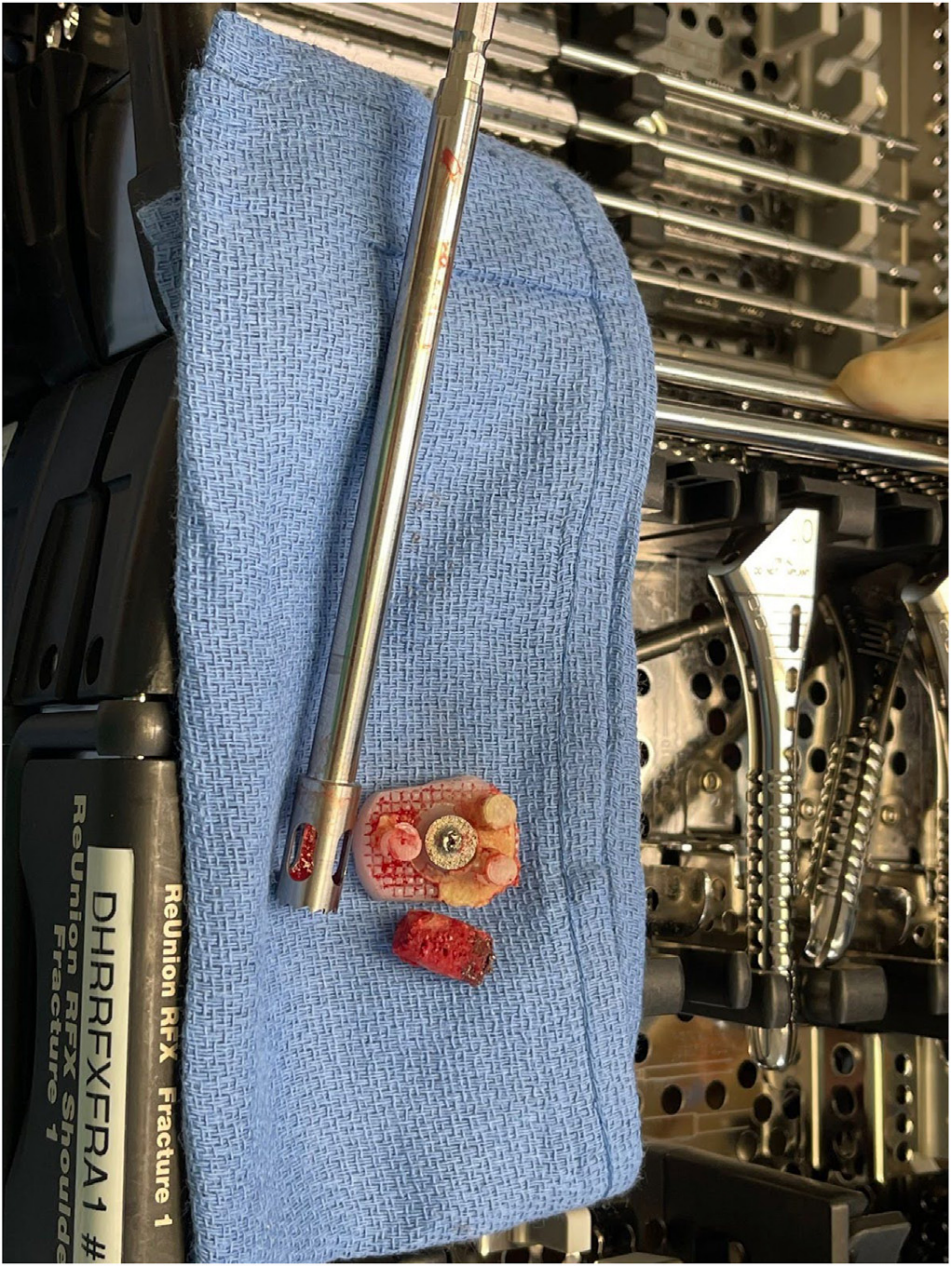
Trabeculated glenoid baseplate with fractured stem and trephine used to remove the stem from the glenoid.

**Figure 10:**
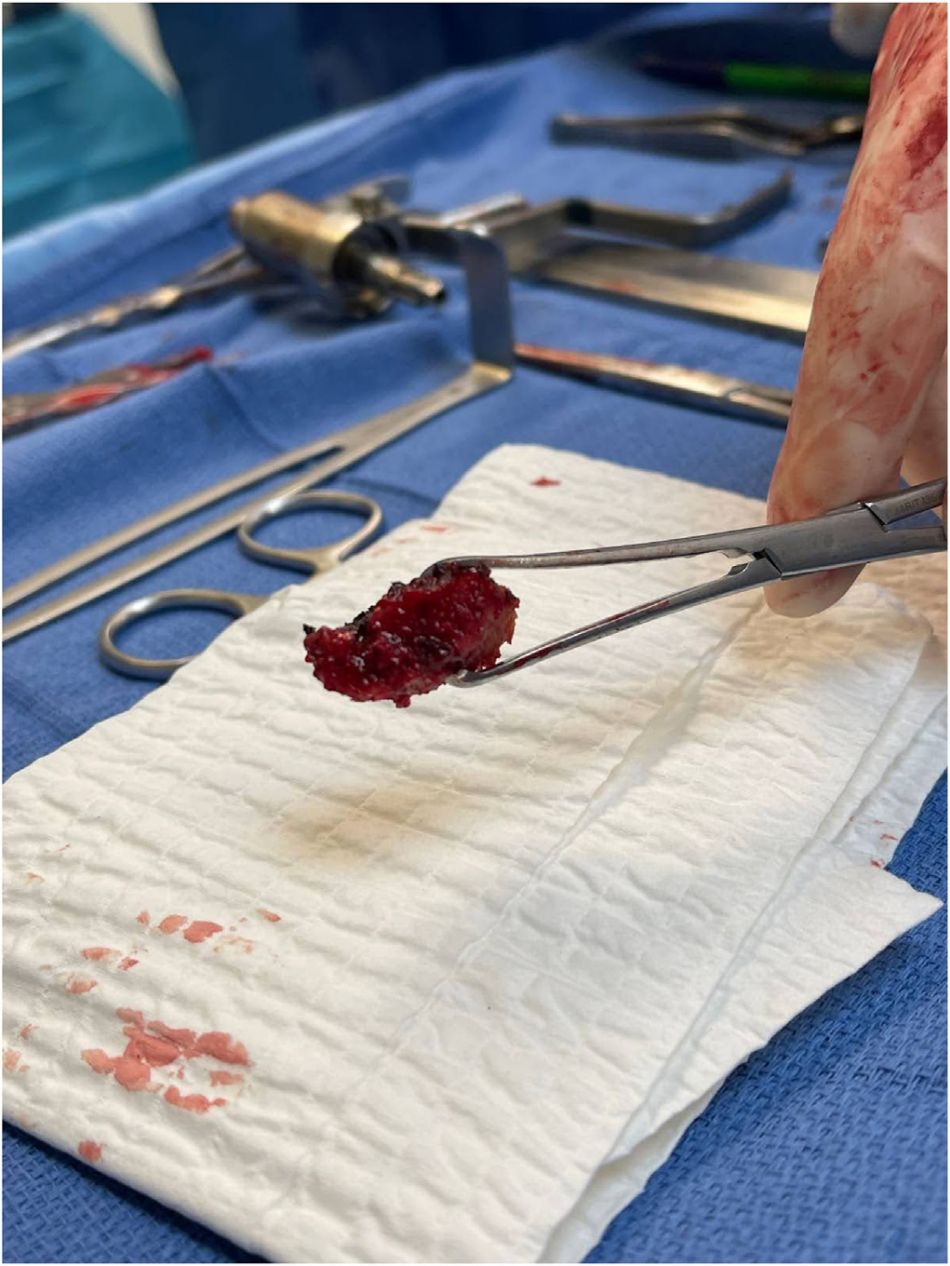
Large central glenoid defect with autologous clavicle graft impaction.

Removal of the shoulder prostheses ensued. Utilizing a tuning fork, the humeral head component was removed from its stem, and the stem was retracted posteriorly for adequate visualization of the glenoid component. The glenoid component was found to be completely mobile and had fractured from the central peg. The central peg was well fixed with bony ingrowth, requiring trephine for removal. Following the central peg and cement removal, a large central glenoid bone defect was present, necessitating impaction grafting. Figure 5, Figure 6 and Figure 5, Figure 6 demonstrate the large central glenoid defect following removal of the glenoid baseplate stem. Figure 7, Figure 8, Figure 9, Figure 10 show the trabeculated glenoid baseplate with a fractured stem and the trephine used to remove the stem from the glenoid.

Upon identifying the large central glenoid bone defect, the deltopectoral incision was extended superiorly to expose the acromioclavicular joint for distal clavicle grafting. Following palpation of the intact coracoclavicular ligaments medially, one centimeter of the distal clavicle was then resected. The resected clavicle was prepared via shaping and removal of cortical bone and cartilage for graft preparation. Gelfoam was used to aid in hemostasis. Following graft preparation, the impaction of the distal clavicle graft into the glenoid bone defect took place with an excellent fit. Glenoid baseplate attachment ensued with a 40 mm central screw, 24 mm screws superiorly and inferiorly, and 16 mm screws anteriorly and posteriorly. This allowed for a 32 + 6 mm glenosphere impaction onto the baseplate in a standard manner.

**Figure 11:**
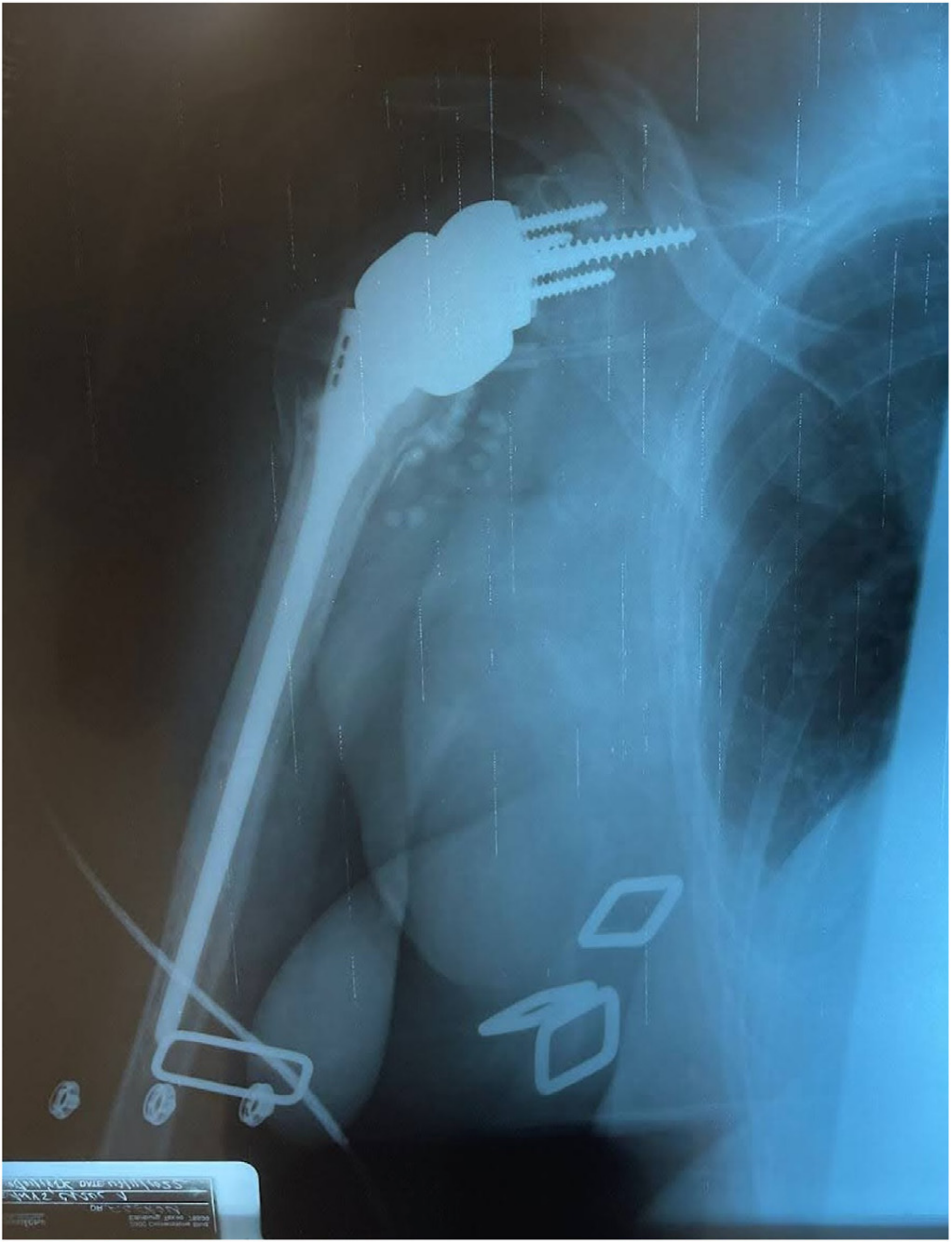
Immediate postoperative radiographs following distal clavicle resection for large central glenoid defect and adequate glenoid baseplate fixation.

Humeral stem and head replacement were performed in standard fashion, utilizing a size 8 stem and 10mm tray with a 4mm cup. The tuberosities were reshaped to ensure proper placement of the implant and enhance their integration with it. Drill tunnels were then created through the tuberosities to allow fiber tape to pass through and securely attach to the implant. Following tuberosity repair, subscapularis and supraspinatus tendons were secured in a standard manner for added stability. Copious irrigation was performed, and although the frozen section and infection work-up were negative, prophylactic antibiotic beads were used due to the revision nature of the case. The incision was then closed in layers. Postoperatively, the patient's right upper extremity was placed in a shoulder immobilizer sling, and she was instructed to follow-up in the clinic one week postoperatively. The immediate postoperative radiograph is seen in Figure 11. The patient was kept overnight for further observation and monitoring before discharge home.

## Results

**Figure 12:**
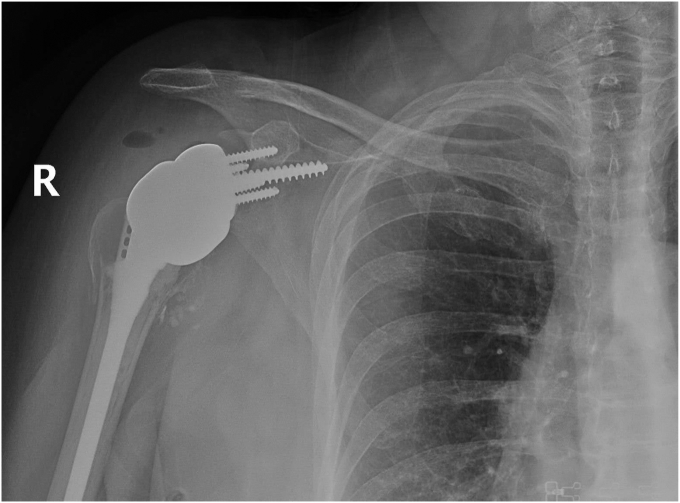
2-week postoperative radiographs following distal clavicle resection for large central glenoid defect and adequate glenoid baseplate fixation.

**Figure 13:**
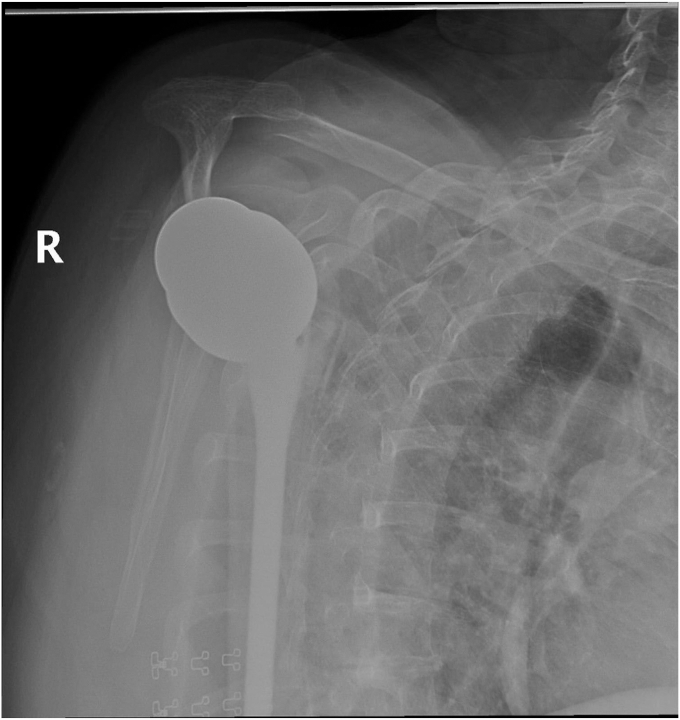
2-week postoperative radiographs following distal clavicle resection for large central glenoid defect and adequate glenoid baseplate fixation.

**Figure 14:**
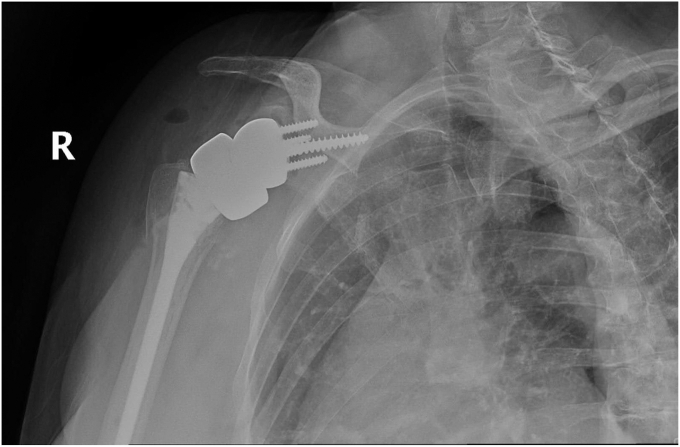
2-week postoperative radiographs following distal clavicle resection for large central glenoid defect and adequate glenoid baseplate fixation.

**Figure 15:**
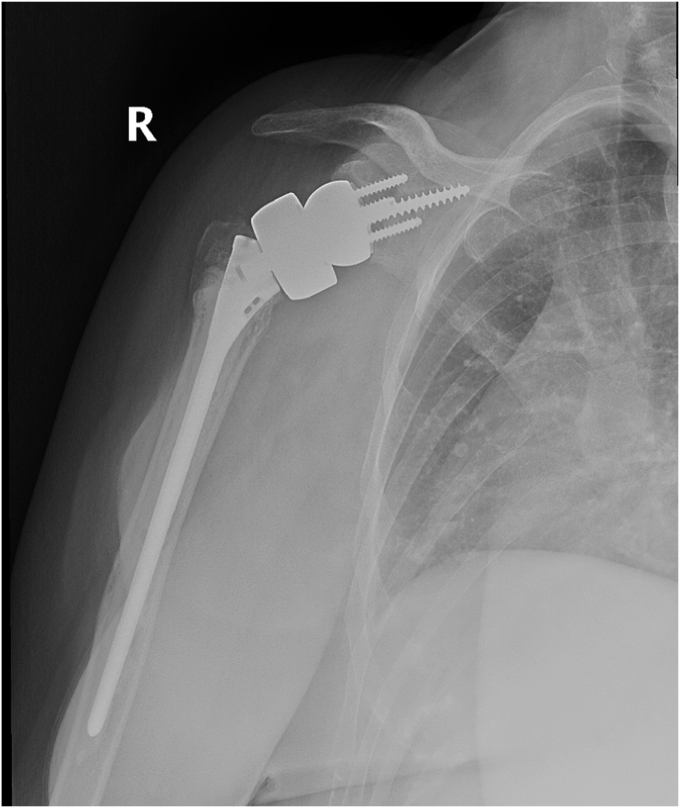
Two-month postoperative follow-up with adequate glenoid baseplate fixation and healing of humeral tuberosities.

Our patient followed up weekly for the first 2 weeks, monthly for the first 3 months, and then 6 months and 1 year postoperatively. The patient’s shoulder was immobilized in a sling with instructions for passive pendulum exercises several times daily for the first 6 weeks. At the 6-week mark, the patient participated in physical therapy for additional strength and mobility, which incrementally increased in intensity under supervision. By 3 months postoperatively, the patient reported no further pain and could complete most ADLs without significant limitations. Active and passive forward flexion had improved to 90 degrees for both. Follow-up radiographs showed adequate glenoid baseplate fixation and healing of the humeral tuberosities, as seen in Figure 12, Figure 13, Figure 14, Figure 15 at two different time frames (two-weeks and two-months). At one year postoperatively, our patient showed no failure of the revision components, had resolution of her pain, and was able to complete all ADLs without limitation.

## Discussion

Despite promising clinical outcomes, revision rTSA involves complexities that can be particularly demanding for surgeons; however, can be an effective treatment option.^19^ The complexity of revision rTSA's is primarily attributed to glenoid bone loss defects and irreparable rotator cuffs. Autologous grafting has become more commonplace in glenoid bone defects, typically utilizing the iliac crest. Other autologous grafting sites for glenoid defects have included the distal tibia, femoral head and neck, coracoid, and clavicle. However, the ability to reconstruct glenoid bone volume for baseplate attachment and restoration of the glenohumeral joint line is complicated by limited exposure, the amount of graft required, and the likelihood of graft uptake.

Choosing an appropriate autologous graft for glenoid bone defects has remained a topic of debate. Iliac crest autografting has been associated with good clinical outcomes in treating large glenoid bone defects, although requires an additional surgical site.^20^ Norris et al described a 1-stage technique of harvesting an iliac crest graft after the glenoid baseplate is secured to the iliac crest, allowing for immediate stability of the glenosphere upon fixation of the bone graft and baseplate to the native scapula.^13^ Other prior studies have demonstrated the viability of distal clavicle grafting for glenoid bone loss in shoulder instability; however, few studies have looked at the viability of distal clavicle grafting in revision TSA specifically.^11^^,^^14^^,^^18^

In our case, distal clavicle resection and grafting for glenoid bone loss appears viable, given the promising preliminary results in the operating room and postoperative follow-up examinations. Using the distal clavicle as an autologous graft gives surgeons a graft readily available in glenoid bone loss defects and is relatively cost-effective. In addition, this allows for a 1-stage technique to correct glenoid defects and perform revision rTSA without having two areas prepped and draped.

It should also be noted using a trabecular metal glenoid base plate in reverse TSA has gained popularity as the baseplate provides stability through a combination of screw fixation and bony ingrowth into the porous baseplate. At five years, Kaplan-Meier survivorship of trabecular metal glenoid baseplates is estimated at 96.7%, with aseptic glenoid failure as the endpoint.^17^ Despite promising survivorship, the trabeculated glenoid baseplates come with challenges in the revision of TSA. Theivendran et al found in their retrospective review of rTSAs that 124 of 125 (99.2%) trabeculated glenoid baseplates had a high rate of glenoid bone integration.^17^ Given the bony ingrowth of the baseplate, removing this component can be extremely difficult and cause further damage to the glenoid. In our case, the glenoid bone integration led to the fracturing of the glenoid component stem and ultimately required the utilization of a trephine to remove the stem from the glenoid.

## Conclusion

This article highlights the use of distal clavicle grafting for reconstructing large central glenoid bone defects in revision shoulder arthroplasty. The technique demonstrated promising short-term outcomes, including improved shoulder function and stability. Although the initial results are encouraging, further follow-up and additional cases are needed to confirm the long-term efficacy and reliability of this approach. The risk of graft failure and subsequent glenoid loosening remains a concern; however, we will continue to follow closely and remain optimistic. Continued monitoring and further clinical studies will be essential to fully assess the viability of distal clavicle grafting and its role in managing glenoid bone loss in revision shoulder arthroplasty.

## Disclaimers:

Funding: No funding was disclosed by the authors.

Conflicts of interest: The authors, their immediate families, and any research foundation with which they are affiliated have not received any financial payments or other benefits from any commercial entity related to the subject of this article.

Patient consent: Obtained.

## References

[1] Antuna S.A., Sperling J.W., Cofield R.H., Rowland C.M. (2001). Glenoid revision surgery after total Shoulder Arthroplasty. J Shoulder Elbow Surg.

[2] Best M.J., Aziz K.T., Wilckens J.H., McFarland E.G., Srikumaran U. (2021). Increasing incidence of primary reverse and anatomic total shoulder arthroplasty in the United States. J Shoulder Elbow Surg.

[3] Buckingham B.P., Parsons I.V.M., Campbell B., Titelman R.M., Smith K.L., Matsen F.A. (2005). Patient functional self-assessment in late glenoid component failure at three to eleven years after total shoulder arthroplasty. J Shoulder Elbow Surg.

[4] Cheung E., Willis M., Walker M., Clark R., Frankle M.A. (2011). Complications in reverse total shoulder arthroplasty. J Am Acad Orthop Surg.

[5] Flurin P.H., Janout M., Roche C.P., Wright T.W., Zuckerman J. (2013). Revision of the loose glenoid component in anatomic total shoulder arthroplasty. Bulletin Hosp Jt Dis.

[6] Formaini N.T., Everding N.G., Levy J.C., Santoni B.G., Nayak A.N., Wilson C. (2015). The effect of glenoid bone loss on reverse shoulder arthroplasty baseplate fixation. J Shoulder Elbow Surg.

[7] Franta A.K., Lenters T.R., Mounce D., Neradilek B., Matsen F.A. (2007). The complex characteristics of 282 unsatisfactory shoulder arthroplasties. J Shoulder Elbow Surg.

[8] Gerber C., Nyffeler R.W. (2002). Classification of glenohumeral joint instability. Clin Orthop Relat Res.

[9] Gupta A., Thussbas C., Koch M., Seebauer L. (2018). Management of glenoid bone defects with reverse shoulder arthroplasty—surgical technique and clinical outcomes. J Shoulder Elbow Surg.

[10] Itoi E., Lee S.-B., Berglund L.J., Berge L.L., An K.-N. (2000). The effect of a glenoid defect on anteroinferior stability of the shoulder after Bankart Repair: A cadaveric study. J Bone Joint Surg Am.

[11] Kwapisz A., Fitzpatrick K., Cook J.B., Athwal G.S., Tokish J.M. (2018). Distal clavicular osteochondral autograft augmentation for glenoid bone loss: A comparison of radius of restoration versus Latarjet Graft. Am J Sports Med.

[12] Lo I.K.Y., Parten P.M., Burkhart S.S. (2004). The inverted pear glenoid: An indicator of significant glenoid bone loss. Arthroscopy.

[13] Norris T.R., Kelly J.D., Humphrey C.S. (2007). Management of glenoid bone defects in revision shoulder arthroplasty: a new application of the reverse total shoulder prosthesis. Tech Shoulder Elbow Surg.

[14] Petersen S.A., Bernard J.A., Langdale E.R., Belkoff S.M. (2016). Autologous distal clavicle versus autologous coracoid bone grafts for restoration of anterior-inferior glenoid bone loss: A biomechanical comparison. J Shoulder Elbow Surg.

[15] Provencher M.T., Bhatia S., Ghodadra N.S., Grumet R.C., Bach B.R., Dewing C.B. (2010). Recurrent shoulder instability: Current concepts for evaluation and management of Glenoid Bone Loss. J Bone Joint Surg Am.

[16] Schairer W.W., Nwachukwu B.U., Lyman S., Craig E.V., Gulotta L.V. (2015). National Utilization of reverse total shoulder arthroplasty in the United States. J Shoulder Elbow Surg.

[17] Theivendran K., Varghese M., Large R., Bateman M., Morgan M., Tambe A. (2016). Reverse total shoulder arthroplasty using a trabecular metal glenoid base plate. Bone Joint J.

[18] Tokish J.M., Fitzpatrick K., Cook J.B., Mallon W.J. (2014). Arthroscopic distal clavicular autograft for treating shoulder instability with glenoid bone loss. Arthrosc Tech.

[19] Walker M., Willis M.P., Brooks J.P., Pupello D., Mulieri P.J., Frankle M.A. (2012). The use of the reverse shoulder arthroplasty for treatment of failed total shoulder arthroplasty. J Shoulder Elbow Surg.

[20] Warner J.J., Gill T.J., O'Hollerhan J.D., Pathare N., Millett P.J. (2006). Anatomical glenoid reconstruction for recurrent anterior glenohumeral instability with glenoid deficiency using an autogenous tricortical iliac crest bone graft. Am J Sports Med.

